# Prevention of Epididymitis

**Published:** 1907-02

**Authors:** 


### PREVENTION OF EPIDIDYMITIS.

Wm. T. Belfield, in the New York Medical Journal, tells how the successful treatment of gonorrheal and other infections of the seminal duct and vesicle by injections through the vas deferens led him to attempt the prevention of the spread of such infections downwards to the epididymis.

Four such attempts have been made; in two by simply opening the canal of the vas and injecting the proximal duct and vesicle with a solution of silver compound; in two others by complete section of the vas and similar injections. In all of these—cases of acute gonorrhea —the symptoms usually preceding descent of the infection to the epididymis, including pain and tenderness in the inguinal canal, were present. No epididymitis occurred in any of them. In two other cases of acute gonorrhea, the infection had already reached the epididymis some twenty-four or thirty-six hours earlier; in each the vas was divided and the vesicle injected, and in each the epididymitis was unusually mild, the pain and swelling remaining so slight that the patients were not confined to bed.

Gonorrheal epididymitis has commonly two serious features: The loss from business of four to ten days' time, and the probability of permanent occlusion of the duct and consequent sterility of the affected side. The exact proportion of this probability cannot, for lack of sufficient data, be confidently stated; estimates vary from fifty to ninety per cent., the latter based upon the observation of Liegeois, who found spermatozoa in the semen of only eight out of eighty-three patients who had had bilateral gonorrheal epididymitis.

Therefore, even if occlusion of the vas were a probable result of this interference, the prevention would be preferable to the epididymitis for two reasons: 1, It prevents loss of time; 2, the occlusion would be in the vas, where subsequent end to end anastomosis is easy and successful, instead of in the epididymis, where anastomosis is impracticable without the technical skill of the originator of that operation, E. Martin.

But the operation that the author performs does not occlude the vas, a statement based upon experimental observations upon dogs, and clinical observation upon a patient (one of the four mentioned) who possessed only one testicle, the other having been removed some years earlier. This man's semen, examined at intervals for six months after the operation, always contained abundant spermatozoa.

The operation is thus performed: The vas is held by the fingers against the skin of the scrotum near the median line, while a half curved needle is passed through the skin under the vas. A half inch incision exposes the vas; a transverse or longitudinal incision into the vas opens its canal. The blunted needle (curved) of a hypodermic syringe can be passed into this minute canal, and a watery solution of any chosen agent injected; this liquid traverses the vas and ampulla, and enters the seminal vesicle. A fine silkworm gut suture is passed into the lumen of the canal at each extremity of the incision and out through the wall of the vas a quarter inch or more distant; one suture end is then passed through the skin and the two ends tied loosely outside. This suture, entering the lumen of the proximal end, serves to buide the needle wneh daily injections are to be made. When complete transverse section of the vas is made, the suture arrangement is identical with that described for the fistula formation. When the incision into the vas is longitudinal, an additional suture (catgut) is passed through the edges and tied loosely above the skin. This operation is done under local anesthesia in the office, the patient walking away at its conclusion and losing no time from his vocation.

When restoration of the canal is desired, the silkworm suture is tightened so as to appose the cut ends; when the wound is healed the suture is removed. Restoration of the lumen of an occluded vas is accomplished by exercising the occluded portion and suturing the divided ends in the same way; the lumen of the vas is maintained during healing by the thread within it.—*Medical Standard, Jan.* '07.

